# Variable-Order Fractional Models for Wall-Bounded Turbulent Flows

**DOI:** 10.3390/e23060782

**Published:** 2021-06-20

**Authors:** Fangying Song, George Em Karniadakis

**Affiliations:** 1 College of Mathematics and Computer Science, Fuzhou University, Fuzhou 350108, China; fysong@stu.xmu.edu.cn; 2 Division of Applied Mathematics, School of Engineering, Brown University, Providence, RI 02912, USA

**Keywords:** fractional conservations laws, variable fractional model, turbulent flows, fractional PINN, physics-informed learning

## Abstract

Modeling of wall-bounded turbulent flows is still an open problem in classical physics, with relatively slow progress in the last few decades beyond the log law, which only describes the intermediate region in wall-bounded turbulence, i.e., 30–50 y+ to 0.1–0.2 R+ in a pipe of radius R. Here, we propose a fundamentally new approach based on fractional calculus to model the entire mean velocity profile from the wall to the centerline of the pipe. Specifically, we represent the Reynolds stresses with a non-local fractional derivative of variable-order that decays with the distance from the wall. Surprisingly, we find that this variable fractional order has a universal form for all Reynolds numbers and for three different flow types, i.e., channel flow, Couette flow, and pipe flow. We first use existing databases from direct numerical simulations (DNSs) to lean the variable-order function and subsequently we test it against other DNS data and experimental measurements, including the Princeton superpipe experiments. Taken together, our findings reveal the continuous change in rate of turbulent diffusion from the wall as well as the strong nonlocality of turbulent interactions that intensify away from the wall. Moreover, we propose alternative formulations, including a divergence variable fractional (two-sided) model for turbulent flows. The total shear stress is represented by a two-sided symmetric variable fractional derivative. The numerical results show that this formulation can lead to smooth fractional-order profiles in the whole domain. This new model improves the one-sided model, which is considered in the half domain (wall to centerline) only. We use a finite difference method for solving the inverse problem, but we also introduce the fractional physics-informed neural network (fPINN) for solving the inverse and forward problems much more efficiently. In addition to the aforementioned fully-developed flows, we model turbulent boundary layers and discuss how the streamwise variation affects the universal curve.

## 1. Introduction

Reynolds [1] was the first to statistically describe turbulence by decomposing the instantaneous velocity vector into an average field and its fluctuation. Upon substitution into the Navier–Stokes equations and averaging, assuming quasi-stationarity, a new modified equation emerged for the average velocity that includes an additional term, namely, the averaged dissipation tensor leading to the turbulence-closure problem [2]. Addressing the closure complexity has been a century-long pursuit, starting with the seminal work of Prandtl [3], who proposed a simplified mixing length model analogous with Fick’s law of local diffusion. Interestingly, at about the same time, Richardson [4], in an attempt to unify turbulent diffusion with molecular diffusion, combined geophysical measurements with Brownian motion to produce the famous scaling law on turbulent pair diffusivity. While ingenious, both approaches assume implicitly locality in turbulent interactions, which limits the universality of the derived correlations—an open standing question for over a century. As stated by Kraichnan [5], Prandtl’s approach is valid only when the spatial scale of inhomogeneity of the mean field is large compared to the mixing length. This assumption is clearly violated in most turbulent flows, e.g., in Reynolds’ pipe flow, where the turbulent eddies are of the size of the pipe radius. This has motivated research in nonlocal constitutive equations of turbulence, and Prandltl, in subsequent work [6], developed a turbulent shear-layer model in an attempt to introduce non-locality in his approach. Kraichnan [5] pioneered such non-local approximations and, based on his work, more recently generalized versions of the second Prandtl non-local model were proposed in the literature [7].

Fractional calculus is an effective tool to solve complex problems with nonlocality and scale-free self-similar processes as well as non-Gaussian statistics. Lévy statistics lead to anomalous diffusion [8] and can effectively model turbulent intermittency [9]. Hence, it is possible that turbulent eddy diffusion could be accurately modeled by fractional Reynolds stresses [10]. Based on physical arguments, in order to represent nonlocality and intermittency, Chen [11] proposed a fractional Laplacian as a model for representing the Reynolds stress with a fixed fractional exponent α=2/3. More recently, starting with the Boltzmann equation, Epps et al. [12] rigorously derived the fractional Navier–Stokes equations by replacing the Maxwell–Boltzmann distribution with the more general Levy α-stable distribution; see a recent extension of this work in [13]. For α=2, the new equations revert to the standard Navier–Stokes equations, while for α=1, we obtain the logarithmic velocity profile known as the law of the wall [14]. The work of Epps et al. [12] laid a new framework for turbulence modeling that may lead to new fundamental understanding of turbulence, but it is only valid in an open domain and thus ignores the important issue of nonlocal boundary conditions encountered in defining fractional Laplacians in bounded domains [15].

The work we include here incorporates our first paper [16] published in the archives, and is a significant extension. We also refer to the work of [17], who modeled the total shear stress directly in wall units by formulating a one-sided variable-order model using the Caputo fractional derivative for Couette flow [17] and in ongoing work on transitional and turbulent boundary layers. For the case of Couette flow, universality was found. We note that directly formulating the problem in wall units does not require modeling of any additional coefficients, unlike the formulation in the present study.

The remainder of this paper is organized as follows: Since the small-scale components can be described as an anomalous diffusion [11], we introduce the variable-order fractional calculus in the next section. Then, we formulate the inverse optimization problem corresponding to the governing equations. We present the fractional differential equations to model different turbulent flows (e.g., channel flow, Couette flow, and pipe flow) in Section 2. The inverse problem is solved by a finite difference (FD) method to obtain the fractional order. Moreover, we introduce the fractional physics-informed neural network for solving the inverse problem to find the variable-orders. In Section 3, we present the numerical results that show that the universal fractional-order profiles of the channel and pipe flow as a function of the distance from the wall, a unique capability enabled by fractional calculus. In particular, we discovered that this fractional-order function is universal for all Reynolds numbers and for different geometries. Finally, we provide a short summary in Section 4.

## 2. Variable-Order Fractional Models for Turbulent Flows

The first fractional model for the Reynolds averaged Navier–Stokes equations was developed by Chen [11], who proposed a fractional Laplacian to model the Reynolds stresses and to account for intermittency [18,19] as follows:

(1) $$\frac{\partial U}{\partial t}+U\cdot \nabla U=-\frac{1}{\rho }\nabla P+\nu_{0}\Delta U-\gamma {\left(-\Delta \right)}^{1/3}U,$$

where *U* is the average velocity and γ is the turbulent diffusion coefficient. Hence, the effective fractional order in this model is fixed at α=2/3. This value is consistent with the Richardson superdiffusion scaling for homogeneous turbulence that leads to a $t^{3}$ scaling for the mean square displacement, but it is not valid for wall-bounded turbulence where anisotropy and the distance from the wall determine the effective rate of turbulent diffusion. Defining a fractional Laplacian in multiple dimensions and in bounded domains is still an open issue in fractional calculus and extending it to variable orders is challenging [15]. However, other somewhat equivalent definitions based on tempered fractional calculus [20] may lead to satisfactory nonlocal representations as well; specifically, in a Boltzmannian framework, Samiee et al. [13] developed a tempered fractional subgrid-scale model to capture high-order structures at the inertial and dissipative ranges. As Richardson first noted, the velocity field in the atmosphere shares a number of properties with the Weierstrass function, i.e., it appears to be continuous but non-differentiable, and this provides a strong case for fractional modeling of turbulence in the atmosphere but also in wall-bounded flows in engineering applications.

In this section, we present a variable-order fractional model for turbulent flows. We firstly consider a one-sided model for channel and pipe flows. Furthermore, we formulate an inverse problem for the fractional order α(y). We present a finite difference method and design a physics-informed neural network (PINN) to obtain the fractional order. Finally, we propose a divergence variable fractional (two-sided) model for turbulent flows.

### 2.1. Turbulent Channel Flow and Pipe Flow

#### 2.1.1. One-Sided Fractional Derivative Modeling

For wall-bounded turbulence, the effective rate of diffusion varies with distance from the wall. Hence, we exploit the power of fractional calculus that allows variable fractional order, and we propose a variable-order fractional differential equation for modeling the Reynolds stresses, i.e., α(y), where *y* is the distance from the wall. In particular, we consider fully developed turbulent flows with one-dimensional (dimensionless) averaged velocity U(y)=u/V (where *V* is the characteristic velocity), including channel flows and pipe flows for which we apply a unified fractional modeling approach. Specifically, assuming that the flow direction is along *x* and *y* is the wall-normal direction (distance from the wall), we consider the variable fractional model (VFM-I) in the normalized interval [0, 1]:

(2) $$\left(\mathrm{VFM}-I\right)\;\;\;\;\frac{\partial }{\partial y}\left(\nu_{0}\frac{\partial U}{\partial y}-\bar{u^{\prime }v^{\prime }}\right)=\nu \left(y\right)D_{y}^{\alpha (y)}U=f,\;\forall y\in \Lambda =\left(0,1\right],$$

with α(0)=1, 0≤α(y)≤1, $D_{y}^{\alpha }$ is the (Caputo) fractional derivative, $f=-\frac{1}{\rho }\partial P/\partial x$ is a constant pressure gradient, U(y) is the mean velocity we want to model, and $\nu_{0}$ is the kinematic viscosity. The Caputo derivative is defined as:

$$D_{y}^{\alpha }U\left(y\right)=\frac{1}{\Gamma (1-\alpha )}\int_{0}^{y}{\left(y-\tau \right)}^{-\alpha }U^{\prime }\left(\tau \right)d\tau ,$$

and it is identical to the Riemann–Liouville left-sided derivative because U(0)=0. Interestingly, we can obtain the scalar coefficient ν(y) (we refer to it as turbulent diffusivity, although it does not have the correct units) explicitly in terms of the fractional order α(y) from:

(3) $$\nu \left(y\right)=f\Gamma \left(2-\alpha \left(y\right)\right)Re_{\tau }^{-\alpha (y)}V/u_{\tau },$$

where $Re_{\tau }=u_{\tau }R/\nu_{0}$ is the friction Reynolds number, *R* is the radius of the pipe (or the half channel width), and $u_{\tau }$ is the wall friction velocity $u_{\tau }=\sqrt{\tau_{w}/\rho }$, where $\tau_{w}{=\mu \partial U/\partial y|}_{y=0}$ is the wall shear stress with μ being the dynamic viscosity.

We discuss an alternative model, where the variable fractional order α(y) is between one and two instead of the VFM-I we presented, where 0<α(y)≤1; this model is analogous to VFM-I and is defined by:

(4) $$\left(\mathrm{VFM}-\mathrm{II}\right)\;\;\;\frac{\partial }{\partial y}\left(\nu_{0}\frac{\partial U}{\partial y}-\bar{u^{\prime }v^{\prime }}\right)=\nu \left(y\right)D_{y}^{\alpha (y)}U=f,\;\forall y\in \Lambda =\left(0,1\right],$$

with α(0)=2, and the variable-order 1≤α(y)≤2 is an unknown function to be determined by the data. The scalar coefficient ν(y) can also be computed from a similar formula as before, i.e.,

(5) $$\nu \left(y\right)=\lim_{y_{0}\to \frac{1}{Re_{\tau }}}\frac{f}{D_{y}^{\alpha (y)}\left(U{|}_{y_{0}}\right)}.$$

#### 2.1.2. Numerical Method

We assume that we know the mean velocity U(y) (also $U^{+}\left(y^{+}\right)$) from the DNS data or experimental results. The VFM-I can be written in the form:

(6) $$\nu \left(y\right)D_{y}^{\alpha (y)}U=f,$$

where $f=-\frac{1}{\rho }\partial P/\partial x$. Since the fractional order α(y) is unknown in Equation (6), we need to solve a nonlinear problem to obtain α(y). Alternatively, we consider the following optimization problem: given *U* and *f*, find the α(y) that satisfies

(7) $$J\left(\alpha \left(y\right)\right)=\underset{\alpha (y)\in S}{\inf}∥\nu \left(y\right)D_{y}^{\alpha (y)}U-f{∥}^{2},$$

where, $S\left(\Lambda \right):=\{0\leq a\left(y\right)\leq 1,a\left(y\right)\in C^{0}\left(\Lambda \right)\}$. If $\alpha^{*}\left(y\right)$ satisfies Equation (6), then we obtain $J(\alpha^{*}\left(y\right))\equiv 0$.

Next, we present a numerical method for solving the optimization problem (7). The fractional derivative is discretized with the finite difference method. Then, the fractional order α(y) can be solved point-by-point; for each point $y_{n}=n\Delta y,\Delta y=1/N,n=1,2,\cdots ,N$, we calculate the fractional derivative $D_{y}^{\alpha (y_{n})}U^{n}$ with the DNS data using the finite difference method [21]

(8) $$D_{y}^{\alpha (y_{n})}U^{n}=\frac{1}{\Gamma (2-\alpha \left(y_{n}\right))}\sum_{j=0}^{n}b_{j}^{n}\frac{U^{n+1-j}-U^{n-j}}{\Delta y^{\alpha (y_{n})}},$$

where $b_{j}^{n}:={\left(j+1\right)}^{\alpha (y_{n})}-j^{\alpha (y_{n})}$ and $U^{n}=U\left(y_{n}\right)$. The discrete optimization problem can now be written as

(9) $$J_{N}\left(\alpha \left(y\right)\right)=\underset{\alpha (y)\in S}{\inf}\sum_{n=1}^{N}|\nu \left(y_{n}\right)D_{y}^{\alpha (y_{n})}U^{n}-f\left(y_{n}\right){|}^{2}\Delta y.$$

**Figure 1:**
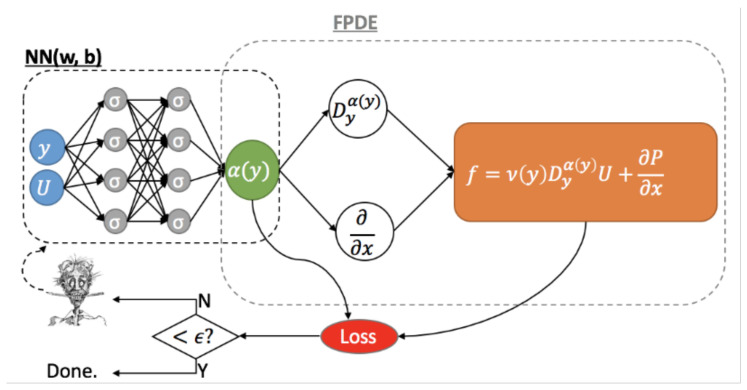
Basic structure of fPINN in 1D for the inverse fractional-order problem. The left uninformed DNN processes data to predict the fractional order, which also has to satisfy the correct physics of turbulence for the channel fully developed flow, represented by the right informed DNN induced by the fractional governing equation.

Finally, we formulate the fractional physics-informed neural network (fPINN) for the inverse problems of the proposed turbulence model; see Figure 1.

The aim of the inverse problem is to estimate the fractional order α(y) given the mean velocity profile *U* in the DNS data. We approximate the variable fractional order α(y) by a multi-layer feedforward neural network $\alpha_{NN}\left(y;\theta ={\left\{W_{j},b_{j}\right\}}_{j=1}^{l}\right)$, where θ are a collection of parameters of the NN. The locations *y* are the input of the NN, and the output *U* is computed by a recursive formula $Y^{j}=\sigma \left(W_{j}Y^{j-1}+b_{j}\right)$ with the initial value $Y^{0}=y$. The weight matrix between the (j−1)th and *j*th layers has the dimension $W_{j}\in R^{n_{j}\times n_{j-1}}$, and the bias vector $b_{j}$ in the *j*th layer. The column vectors $Y^{j-1}\in R^{n_{j-1}\times 1}$ and $Y^{j}\in R^{n_{j}\times 1}$ denote the input and output of the *j*th layer, respectively. The input vector $Y^{j-1}$ is first subject to a linear transformation and then an element-wise nonlinear function σ(·), which is called the activation function. The NN consists of one input layer (j=0), l−1 hidden layers (*j* = 1, 2, ⋯,l−1), and one output layer (j=l). The depth of the NN is *l*, and the width of the *j*th layer is $n_{j}$. To determine the parameters θ, we minimize the following loss function with respect to θ

(10) $$L\left(\theta \right)=\frac{1}{N_{t}}\sum_{i=1}^{N_{t}}{\left(D_{y}^{\alpha_{NN}\left(y_{i};\theta \right)}U\left(y_{i}\right)-1\right)}^{2}+{\left(\alpha_{NN}\left(0;\theta \right)-1\right)}^{2},y_{i}\in \left(0,1\right].$$

The first term on the right-hand side is the equation residual, and the second term is the constraint on the fractional order at the wall, i.e., α(0)=1. We select $N_{t}$ training points, ${\left\{y_{i}\right\}}_{i=1}^{N_{t}}$, to enforce the equation residual on them to be zero. The fractional derivative is evaluated using the finite difference method (8). We optimize the loss function with respect to θ, employing a stochastic gradient descent, Adam, written in TensorFlow. Finally, we estimate the variable fractional order using $\alpha_{NN}\left(y;\theta \right)$.

### 2.2. Two-Sided Turbulent Channel Flow

#### 2.2.1. Fractional Modeling in Divergence Form

We consider the Reynolds averaged momentum equation for incompressible fully developed channel flow; the governing equation is as follows

(11) $$\frac{\partial }{\partial y}\left(\nu_{0}\frac{\partial U}{\partial y}-\bar{u^{\prime }v^{\prime }}\right)+\frac{1}{\rho }\frac{\partial P}{\partial x}=0,\;y\in \left(0,2\right),$$

where ρ is the density; and *P* and *U* are the mean pressure and velocity, respectively. The process of Reynolds averaging introduces the unclosed Reynolds stress, $\tau_{ij}=-\rho \bar{u^{\prime }v^{\prime }}$. The total shear stress on the wall is $\tau_{w}$. Integrating the above equation from wall to an arbitrary position in wall-wise *y*, we obtain a new formula as follows

(12) $$\nu_{0}\frac{\partial U}{\partial y}-\bar{u^{\prime }v^{\prime }}=\tau_{w}/\rho -\frac{1}{\rho }\frac{\partial P}{\partial x}y.$$

We assume the dimensionless wall shear $\tau_{w}$ and pressure gradient $\frac{\partial P}{\partial x}=C$ are constants. Additionally, we introduce a symmetric divergence variable fractional model for approximating the total shear stress,

(13) $$\left(\mathrm{DVFM}\right)\;\;\;\nu_{0}\frac{\partial U}{\partial y}+\bar{u^{\prime }v^{\prime }}=\nu \left(y\right)D_{\left|y\right|}^{\alpha (y)}U=1-y,$$

with the boundary conditions α(0)=α(2)=1, where the fractional derivative is defined as follows

(14) $$D_{\left|y\right|}^{\alpha (y)}U=\frac{1}{2}\left(D_{y}^{\alpha (y)}U+{}_{y}D^{\alpha (y)}U\right),$$

and $D_{y}^{\alpha (y)}$ and ${}_{y}D^{\alpha (y)}U$ are left and right Caputo derivatives, respectively. The definitions are given as follows

$$\mathrm{Left}\;\mathrm{Caputo}\;\mathrm{derivative}:\;D_{y}^{\alpha }U\left(y\right)=\frac{1}{\Gamma (1-\alpha )}\int_{0}^{y}{\left(y-\tau \right)}^{-\alpha }U^{\prime }\left(\tau \right)d\tau ,$$

and

$$\mathrm{Right}\;\mathrm{Caputo}\;\mathrm{derivative}:\;{}_{y}D^{\alpha }U\left(y\right)=-\frac{1}{\Gamma (1-\alpha )}\int_{y}^{2}{\left(\tau -y\right)}^{-\alpha }U^{\prime }\left(\tau \right)d\tau ,$$

and it is identical to the Riemann–Liouville derivatives because U(0)=0 and U(2)=0. We also propose the eddy viscosity in the fractional momentum equation, and the explicit formula is as follows

(15) $$\nu \left(y\right)=\Gamma \left(2-\alpha \left(y\right)\right)Re_{\tau }^{-\alpha (y)},$$

where $Re_{\tau }=u_{\tau }R/\nu_{0}$ is the friction Reynolds number, *R* is the radius of the pipe (or the half channel width), and $u_{\tau }$ is the wall friction velocity, $u_{\tau }=\sqrt{\tau_{w}/\rho }$, where $\tau_{w}{=\mu \partial U/\partial y|}_{y=0}$ is the wall shear stress with μ being the dynamic viscosity.

#### 2.2.2. Numerical Method

We assume that we know the mean velocity U(y) (also $U^{+}\left(y^{+}\right)$) from the DNS data or experimental results. Since the fractional order α(y) is unknown in Equation (13), we need to solve a nonlinear problem to obtain α(y). Alternatively, we consider the following optimization problem: given *U* and *f*, find α(y) that satisfies

(16) $$J\left(\alpha \left(y\right)\right)=\underset{\alpha (y)\in S}{\inf}{∥\nu \left(y\right)D_{\left|y\right|}^{\alpha (y)}U-f∥}^{2},$$

where f=1−y and $S\left(\Lambda \right):=\{0\leq a\left(y\right)\leq 1,a\left(y\right)\in C^{0}\left(\Lambda \right)\}$. If $\alpha^{*}\left(y\right)$ satisfies Equation (13), then we obtain $J(\alpha^{*}\left(y\right))\equiv 0$.

Next, we present a numerical method for solving the optimization problem (16). The fractional derivative is discretized with the finite difference (FD) method. Then, the fractional order α(y) can be solved point-by-point; for each point $y_{n}=n\Delta y$, Δy=1/N, n=1,2,⋯,N, we calculate the fractional derivatives $D_{\left|y\right|}^{\alpha (y_{n})}U^{n}$ with the DNS data using the finite difference method [21]

(17) $$\mathrm{Left}:\;D_{y}^{\alpha (y_{n})}U^{n}=\frac{1}{\Gamma (2-\alpha \left(y_{n}\right))}\sum_{j=0}^{n}b_{j}^{n}\frac{U^{n+1-j}-U^{n-j}}{\Delta y^{\alpha (y_{n})}},$$

and

(18) $$\mathrm{Right}:\;{}_{y}D^{\alpha (y_{n})}U^{n}=-\frac{1}{\Gamma (2-\alpha \left(y_{n}\right))}\sum_{j=0}^{N-n+1}c_{j}^{n}\frac{U^{N-j}-U^{N-j-1}}{\Delta y^{\alpha (y_{n})}},$$

where $b_{j}^{n}:={\left(j+1\right)}^{\alpha (y_{n})}-j^{\alpha (y_{n})}$, $c_{j}^{n}=b_{j}^{n}$ and $U^{n}=U\left(y_{n}\right)$.

The discretized optimization problem can be now written as

(19) $$J_{N}\left(\alpha \left(y\right)\right)=\underset{\alpha (y)\in S}{\inf}\sum_{n=1}^{N}|\nu \left(y_{n}\right)D_{\left|y\right|}^{\alpha (y_{n})}U^{n}-f\left(y_{n}\right){|}^{2}\Delta y.$$

Here, we use $N\approx Re_{\tau }$ points to solve the above optimization for the channel flow at a given Reynolds number $Re_{\tau }$.

Alternatively, we propose the fractional fPINN for solving the inverse DVFM with the loss function

(20) $$L\left(\theta \right)=\sum_{n=1}^{N_{t}}|\nu \left(y_{n}\right)D_{\left|y\right|}^{\alpha_{NN}\left(y_{n};\theta \right)}U^{n}-f\left(y_{n}\right){|}^{2}+|\alpha_{NN}\left(0;\theta \right){-1|}^{2}+{\left|\alpha_{NN}\left(2;\theta \right)-1\right|}^{2}.$$

### 2.3. Turbulent Boundary Layer and Couette Flow

For a boundary layer and Couette flow with zero pressure gradient, the mean two-dimensional continuity and stream-wise momentum reduce to

(21) $$\frac{\partial UU}{\partial x}+\frac{\partial VU}{\partial y}=\frac{\partial }{\partial y}\left(\nu_{0}\frac{\partial U}{\partial y}-\bar{u^{\prime }v^{\prime }}\right).$$

If we assume that the convective effects are small near the wall for the boundary layer problem, then the above equation reduces to

(22) $$\frac{\partial }{\partial y}\left(\nu_{0}\frac{\partial U}{\partial y}-\bar{uv}\right)=0.$$

Here, *U* is viewed as a function of *y* due to $\frac{\partial U}{\partial x}=0.$ Since the two plates are infinitely long for the Couette flow, the flow properties cannot change with *x* and all partial derivatives with respect to *x* vanish. Flow motion only occurs in the *x* direction, and thus, V=0. After simplifying the RANS equations, the turbulent Couette flow is governed by Equation (22) too.

Further integrating the above equation provides

(23) $$\nu_{0}\frac{\partial U}{\partial y}-\bar{u^{\prime }v^{\prime }}=C,$$

where *C* is a constant and $\bar{uv}=0$ at the wall, while $\nu \frac{\partial U}{\partial y}$ is simply the wall shear stress $\tau_{w}/\rho$. Then, we have the following equation

$$\left(\mathrm{TCM}\right)\;\;\;\nu \left(y\right)D_{y}^{\alpha (y)}U=\frac{\tau_{w}}{\rho },$$

with α(0)=1, 0<α≤1, $D_{y}^{\alpha }$ is the (Caputo) fractional derivative, and ν(y) is the eddy viscosity defined as

$$\nu \left(y\right)=\Gamma \left(2-\alpha \left(y\right)\right)Re_{\tau }^{-\alpha (y)}.$$

#### Numerical Method

We solve the fractional order α(y) for the turbulent boundary layer problem and Couette flowy using fPINN (Figure 1) with the loss function

$$\begin{array}{ccc} L(\theta ) & = & \sum_{k=0}^{N_{t}}{\left(\nu \left(y_{k}\right)D_{y}^{\alpha_{NN}\left(y_{k};\theta \right)}-\frac{\tau_{w}}{\rho }\right)}^{2}+{\left(\alpha_{NN}\left(0;\theta \right)-1\right)}^{2}\\ & = & \sum_{k=1}^{N_{t}}{\left(Re_{\tau }^{-\alpha_{NN}\left(y_{k};\theta \right)}\sum_{j=0}^{k}\frac{b_{j}^{k}}{\Delta y^{\alpha_{NN}\left(y_{k};\theta \right)}}\left(U^{k+1-j}-U^{k-j}\right)-\frac{\tau_{w}}{\rho }\right)}^{2}+{\left(\alpha_{NN}\left(0;\theta \right)-1\right)}^{2}, \end{array}$$

where *U* is the DNS data. It changes with $Re_{\theta }$ for the boundary layer problem, so there is (implicit) *x* dependence as well.

## 3. Numerical Results

In this section, we present the results for the turbulent channel, pipe, Couette, and boundary layer flows.

### 3.1. Channel Flow

#### 3.1.1. Numerical Results of the One-Sided Models

**Figure 2:**
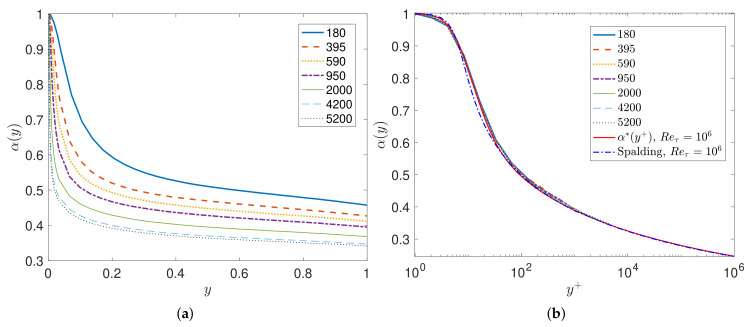
Channel flow modeled with VFM-I: Learning the fractional variable order α(y) using DNS databases at $Re_{\tau }=180\;\mathrm{to}\;5200$: (**a**) profiles of the fractional order α(y); (**b**) rescaled fractional order $\alpha (y^{+})$ in viscous units.

**Figure 3:**
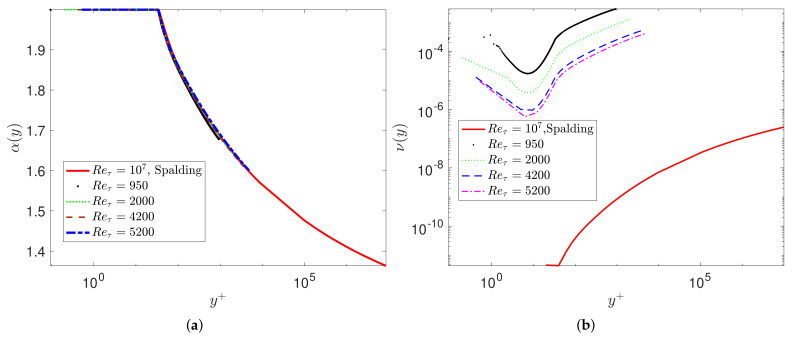
Alternative fractional modeled with VFM-II with 1≤α(y)≤2. The numerical fractional orders are computed based on DNS data for turbulent channel flow at $Re_{\tau }=950,\;2000,\;4200,\;5200$: (**a**) plots of the fractional orders $\alpha (y^{+})$ in wall units; (**b**) corresponding eddy viscosity coefficients.

We first consider turbulent channel flow for which DNS data are available up to $Re_{\tau }=5200$ [22]. Here, we use the FD scheme with $N\approx Re_{\tau }$ points to solve the aforementioned inverse problem for the channel flow at a given Reynolds number $Re_{\tau }$. Solving for α(y), which uniquely determines the Reynolds stresses, Figure 2a depicts the profiles of the fractional order α(y) for different $Re_{\tau }$ as a function of the non-dimensional distance from the wall y∈[0,1]. We see a strong dependence of α(y) on $Re_{\tau }$; however, if we re-plot all data in terms of the viscous wall units, i.e., $y^{+}=yu_{\tau }/\nu_{0}$ we see a collapse of all results into a single universal curve, as shown in Figure 2b. Moreover, we employ the empirical Spalding formula [23] for $U^{+}=u/u_{\tau }$ in order to extend the results up to high $Re_{\tau }=10^{6}$, and again we obtain a similar universal scaling with the exception of low $Re_{\tau }$ for which the Spalding formula is known to be somewhat inaccurate. We fit the fractional order using these numerical results to obtain the fractional order $\alpha (y^{+})$ in wall units as follows

(24) $$\begin{array}{c} \alpha^{*}\left(y^{+}\right)=\frac{1-\phi (y^{+})}{2}+\frac{\phi (y^{+})+1}{2}a(y^{+}), \end{array}$$

where $\phi \left(y^{+}\right)=\tanh\left(\ln\left(y^{+}/9.5\right)/1.049\right)$ and $a\left(y^{+}\right)=1/(b+\kappa |\ln\left(y^{+}\right){|}^{0.9})$ with b=0.855, κ=0.301 are constants. This is a remarkable result as it goes beyond the logarithmic profile and seamlessly connects the viscous sublayer with the buffer zone, the logarithmic profile, and the wake region. Although at first it appears to be a perfect fitting exercise, it has important consequences due to the nonlocal interpretation of the fractional derivative involved, i.e., it shows that nonlocality is stronger away from the wall and at high Reynolds numbers. Using the same data for U(y), we show that the alternative model VFM-II with 1≤α(y)≤2 also leads to the same type of universality (Figure 3). However, unlike the aforementioned VFM-I, we are unable to obtain an explicit formula for ν(y), relating it to the Reynolds number as in the first model (i.e., α(y)∈(0,1]); instead, we can compute it numerically from the DNS data of turbulent channel flow. As shown in Section 3, this alternative fractional model also exhibits a universal scaling if plotted in terms of wall units, with the lowest value of $\alpha (10^{5}+)\approx 1.3$.

**Figure 4:**
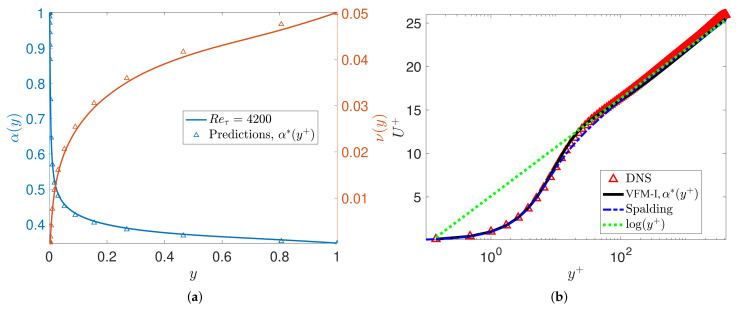
VFM-I: Model predictions for the turbulent channel flow at $Re_{\tau }=4200$: (**a**) the solid line (−) represents the numerical solution of the optimization problem and the triangle symbols (△) represent Equation (24). The blue line represents the fractional order α(y) and the red line is the eddy viscosity coefficient. This Reynolds number $Re_{\tau }=4200$ is not included in the training of the model; (**b**) mean velocity obtained by VFM-I corresponding to the fractional order $\alpha^{*}\left(y^{+}\right)$ from the left plot.

**Figure 5:**
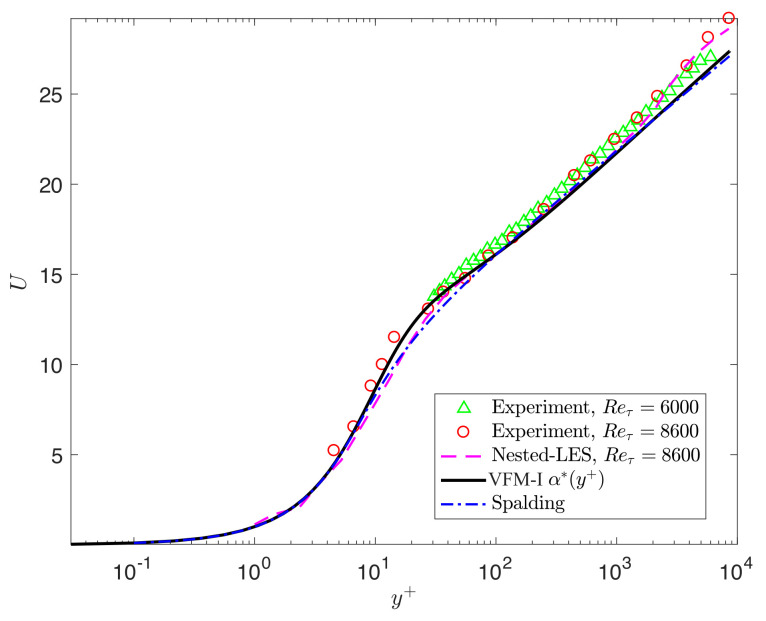
VFM-I: Profiles of the mean velocity for turbulent channel flow at $Re_{\tau }=6000,8600$: the triangle symbol (△) represents experimental data from [25], the circle symbol (∘) represents experimental data from [26], the solid line (−) represents the VFM-I profile, and the dashed line (−−) represents the LES results [24].

To evaluate the predictability of the universal scaling, we now solve the forward Equation (2) to obtain U(y) at $Re_{\tau }=\left[4200,6000,8600\right]$, which are cases not used in the training of the model for $\alpha (y^{+})$. The results presented in Figure 4 and Figure 5 are in good agreement with DNS and experimental data. We also include the turbulent channel flow results obtained by nested LES [24]. Figure 4 and Figure 5 show that the mean velocity profiles predicted by VFM-I exhibit the correct behavior throughout the channel for Reynolds numbers up to $Re_{\tau }=8600$, including the correct slope in the logarithmic layer, and agree with DNS and experimental data in the wake region for all $Re_{\tau }=\left[4200,6000,8600\right]$.

**Figure 6:**
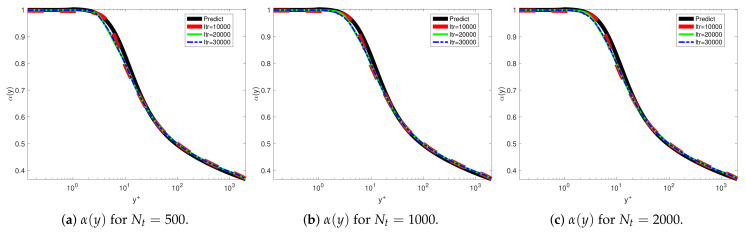
VFM-I: The fractional order obtained from fPINN and from the universal formula derived using point-by-point minimization (“Predict”, Equation (24) ). The training results for the uniform training sets at iteration steps Itr = 10,000, 20,000, 30,000: (**a**) for $N_{t}=500$; (**b**) for $N_{t}=1000$; (**c**) for $N_{t}=2000$.

**Figure 7:**
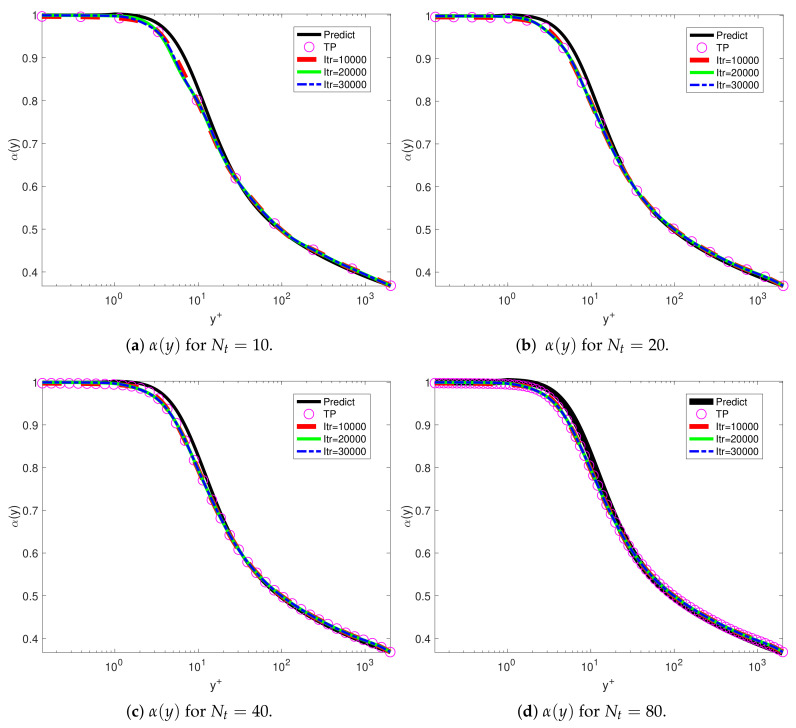
VFM-I: Fractional order for uniform training sets at iteration steps Itr = 10,000, 20,000, 30,000 for different $N_{t}=10,20,30,40$. “Predict” presents the profiles from Equation (1). The friction Reynolds number $Re_{\tau }=2000$. TP, the distribution of the log-uniform training points.

We used fPINN to investigate the turbulent channel flows. We used different training points for investigating the convergence using DNS data at $Re_{\tau }=2000$. Figure 6 shows the training results with uniform training points in the interval for $N_{t}=500,1000,2000$. Figure 7 shows the training results with log-uniform training points in wall units scaling for $N_{t}=10,20,40,80$. The corresponding loss histories are listed in Table 1. Figure 7 presents the comparison profiles between the training sets. We can observe that the results trained by the log-uniform are smoother than the uniform training points near the wall.

Next, we test the accuracy of the forward problem and the loss function error with the training fractional order predicted by log-uniform training points $N_{t}=20$. We solve the fractional equation as follows:

(25) $$\nu \left(y\right)D_{y}^{\alpha (y)}U=f,\;\forall y\in \left(0,1\right],$$

with U(0)=0, and the fractional orderwasis obtained by training fPINN with $N_{t}=20$ and Equation (24). The corresponding loss functional error is defined as follows

$$L\left(\theta \right)=\sum_{k=1}^{N_{t}}{\left(Re_{\tau }^{-\alpha (y_{k})}\sum_{j=0}^{k}\frac{b_{j}^{k}}{\Delta y^{\alpha (y_{k})}}\left(U^{k+1-j}\left(\theta \right)-U^{k-j}\left(\theta \right)\right)-f_{k}\right)}^{2}+{\left(U\left(0;\theta \right)\right)}^{2}.$$

**Figure 8:**
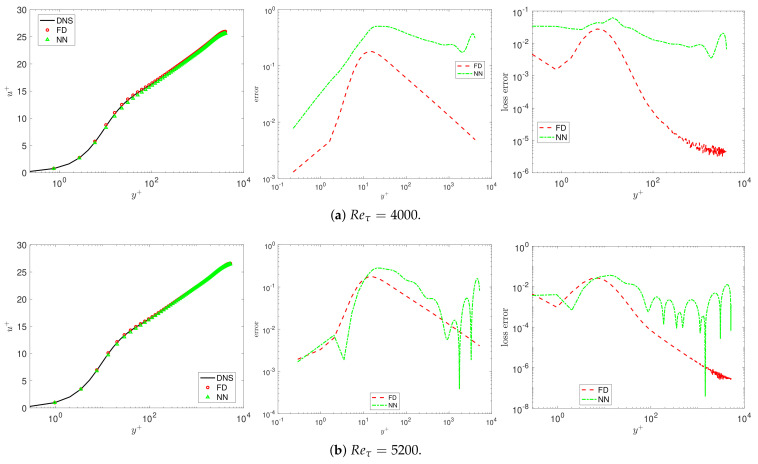
VFM-I: The mean velocity (**left**) for different Reynolds numbers, the pointwise errors of the mean velocity between predictor and DNS data (**middle**), and the loss function (**right**). FD, the fractional order solved by the finite difference method; NN, the results from the neural network.

Figure 8 plots the pointwise error of the mean velocity and the loss function for $Re_{\tau }=4000$ and 5000.

Finally, we use the simplified one-dimensional equation

(26) $$\begin{array}{c} \frac{\partial }{\partial y}\left(\tau_{uv}-R_{uv}\right)=\nu \left(y\right)D_{y}^{\alpha (y)}U=\frac{\partial P}{\partial x},\;y\in \left(0,1\right), \end{array}$$

where the $R_{uv}$ denotes the Reynolds stress $R_{uv}=\bar{u^{\prime }v^{\prime }}$, $\tau_{uv}$ denotes the viscous shear stress $\tau_{uv}=\nu_{0}\partial U/\partial y$, and *U* is the mean velocity, which is the solution to the above fractional Equation (26). Then, we obtain the Reynolds stresses by integration,

(27) $$\begin{array}{c} -R_{uv}=\int_{y}^{1}\nu \left(s\right)D_{s}^{\alpha (s)}Uds-\tau_{uv}. \end{array}$$

**Figure 9:**
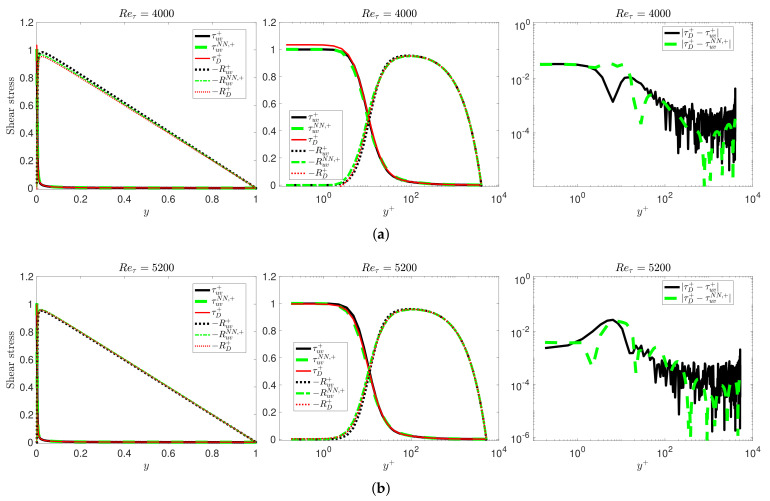
VFM-I: Accurate prediction of the shear stress at (**a**,**b**) $Re_{\tau }=4000,\;5200$ in outer units and wall units: (**left**) outer scaling; (**middle**) wall units scaling; (**right**) pointwise error of the wall shear stress. Here, $\tau_{uv}$ denotes the wall shear stress for the fractional order predicted by the finite difference (FD) method, $\tau_{uv}^{NN}$ denotes the wall shear stress predicted by the NN, and $\tau_{D}$ is the corresponding profile from DNS data. $-R_{uv}$ denotes the Reynolds shear stress predicted by Equation (24), $-R_{uv}$ denotes the wall shear stress predicted by the NN, and $-R_{D}$ is the corresponding profile from DNS data.

We can compare the predicted Reynolds stresses to their counterparts, $R_{D}$ from DNS data for turbulent channel flow, and using the corresponding viscous shear stress denoted by $\tau_{D}=\mu \partial U_{D}/\partial y$, where $U_{D}$ denotes the mean velocity from the DNS database. In Figure 9, we plot the predicted and DNS profiles for Reynolds numbers $Re_{\tau }=4000,\;5200$ and the corresponding pointwise error. We can observe that they are all in very good agreement. The numerical results of the mean velocities and shear stresses for all Reynolds number $Re_{\tau }$ match very well with the DNS data; here, we only show the high Reynolds number cases due to space limitations.

#### 3.1.2. Numerical Results of the Two-Sided Models

**Figure 10:**
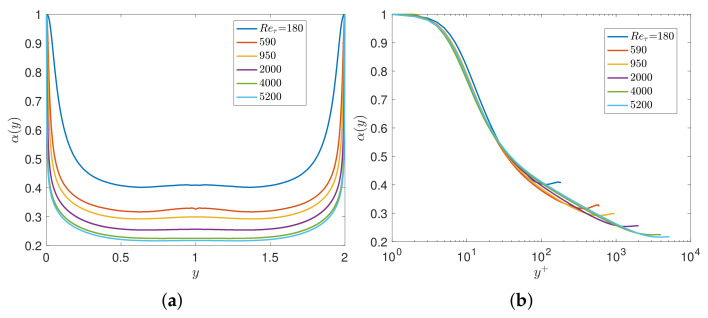
Learning the fractional variable-order α(y) using DNS data bases at $Re_{\tau }=180\;\mathrm{to}\;5200$: (**a**) profiles of the fractional order α(y); (**b**) rescaled fractional order $\alpha (y^{+})$ in viscous wall units.

In this subsection, we focus on the two-sided models. Solving for α(y), which uniquely determines the total shear stresses, Figure 10 plots the profiles of the fractional order α(y) for different $Re_{\tau }$ as a function of the non-dimensional distance between the two walls y∈[0,2]. We see a strong dependence of α(y) of $Re_{\tau }$, which is the same conclusion as for the previous variable fractional model. Furthermore, we re-plot all data in terms of the viscous wall units, i.e., $y^{+}=yRe_{\tau }$, and we see an approximate collapse of all results into a single universal curve in the half-plane excluding the wake region (i.e., near the centerline), as shown in Figure 10.

**Figure 11:**
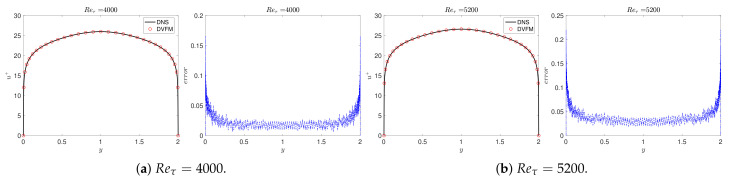
The mean velocity (**left**) and the pointwise difference between the numerical solution and the DNS data (**right**) in each sub-figure.

Next, we test the accuracy of the forward problem with the fractional order provided by the inverse optimization problem (19). We solve the divergence variable fractional equation as follows

(28) $$-D(\nu \left(y\right)D_{\left|y\right|}^{\alpha (y)}U)=1,\forall y\in \left(0,2\right),$$

with U(0)=U(2)=0. Figure 11 plots the solutions (left) of the above equation and the pointwise error (right) of the mean velocity in each subfigure for several $Re_{\tau }$. We can observe that this model predicts the mean velocity well. Moreover, it can obtain a smooth mean velocity profile in the whole domain along the wall-wise direction.

We also use fPINN (20) to solve the inverse problem to obtain the variable order α(y). The two results from the two different methods (i.e., FD and fPINN) agree well for all Reynolds numbers.

### 3.2. Turbulent Pipe Flow

**Figure 12:**
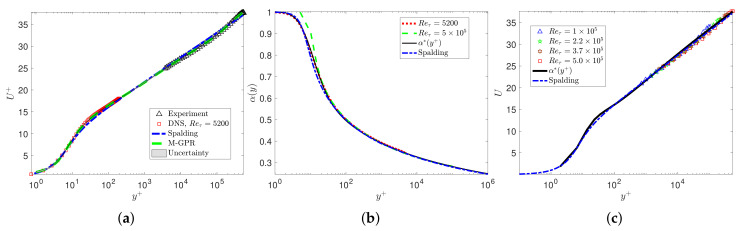
Predictions of the mean velocity profile for the superpipe flow from $Re_{\tau }=1\times 10^{5}\;\mathrm{to}\;5\times 10^{5}$: (**a**) velocity profile reconstructed from the experimental data (△,^28^]), DNS data at $Re_{\tau }=5200$ (□,^22^]), and the Spalding profile (blue line [23]) using multifidelity Gaussian process regression (M-GPR); (**b**) “- -”, fractional order with the M-GPR profile at $Re_{\tau }=5\times 10^{5}$; “-”, the profile of Equation (24); and ‘-·’, the corresponding Spalding profile; (**c**) velocity profiles solving the forward fractional model and the Spalding curve against the experimental data.

**Figure 13:**
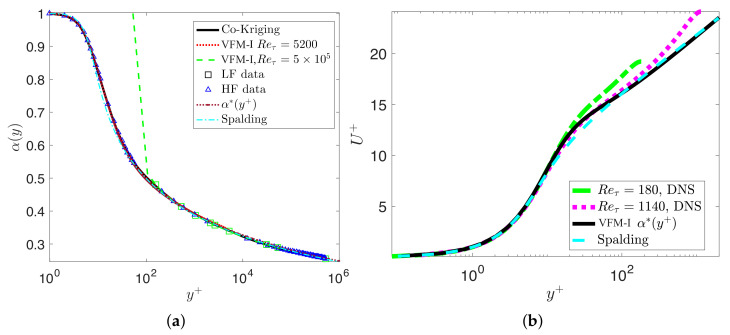
VFM-I for turbulent pipe flow: (**a**) “··”, VFM-I model with the channel flow DNS data at $Re_{\tau }=5200$; “- -”, VFM-I model with the M-GPR profile at $Re_{\tau }=5\times 10^{5}$; “-”, the profile of Equation (24); and ‘-·’, the corresponding Spalding profile; (**b**) ‘-·’ and ‘··’ plot the DNS data at $Re_{\tau }=180$ and $Re_{\tau }=1140$; ‘-’ the VFM-I model at $Re_{\tau }=2000$ and the corresponding Spalding profile.

In this subsection, we consider turbulent pipe flow and again test the universal variable fraction order $\alpha (y^{+})$ against DNS and experimental data. First, we examine the highest Reynolds number available from the superpipe experiment [27,28] at $Re_{\tau }=5\times 10^{5}$, estimated at $Re_{R}\approx 3.525\times 10^{7}$ based on the pipe radius *R*. As the experimental data were only available for $y^{+}>$ 10,000, we synthesized an entire profile from the pipe wall to centerline using multifidelity Gaussian process regression (M-GPR) [29] as follows: we considered as high fidelity data the superpipe data in the outer region together with the highest DNS data for channel flow at $Re_{\tau }=5200$. We then employed the Spalding curve to provide the low-fidelity data and, using M-GPR, we constructed the final profile as shown in Figure 12a. Having this profile and the VFM-I model transformed in polar coordinates, we can then solve the inverse problem and obtain a new variable fractional order $\alpha (y^{+})$. Figure 13a shows that the variable fractional order we obtain for this problem is identical to the function defined by Equation (24). This finding further confirms the universality of the variable fractional order even at very high Reynolds numbers. Having validated the accuracy of the variable fractional order, we can now solve the forward fractional differential problem to obtain predictions of the entire velocity profiles from $Re_{\tau }=10^{5}$ to $Re_{\tau }=5\times 10^{5}$. Figure 12b plots the results, showing that there is excellent agreement with all available data from the superpipe experiment. Figure 13b plots the mean velocity profiles from the DNS data base [30] at low Reynolds numbers, the corresponding VFM predictions, and the Spalding profile. The universal defect law for pipe flows is not valid for the low Reynolds number range, and this is also in agreement with [27], who argued that the lowest $Re_{\tau }$ for universality is approximately 5000.

### 3.3. Turbulent Couette Flow

In reference [12], the authors proposed the double-log profile to predict the mean velocity for the Couette flow as follows

(29) $$\begin{array}{c} U\left(y\right)=\frac{1}{2}-\frac{1}{2}\frac{\ln\left((d+y)/(d+1-y)\right)}{\ln\left(d/(d+1)\right)}, \end{array}$$

where *d* is a small number (d≪1) that represents a viscous sublayer or roughness height. The non-dimensional boundary conditions are U(0)=0 and U(1)=1.

**Figure 14:**
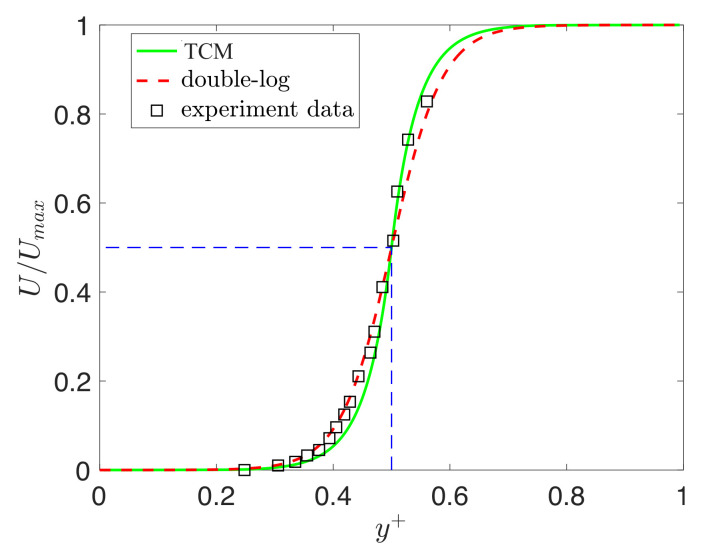
Turbulent Couette flow—numerical results for Re = 16,500: “-”, TCM predictions at $Re_{\tau }=1650$; “- -”, best fit of the double-log profile in Equation (29) with $d=1.06\times 10^{-5}$; “□”, experimental data from [33].

**Figure 15:**
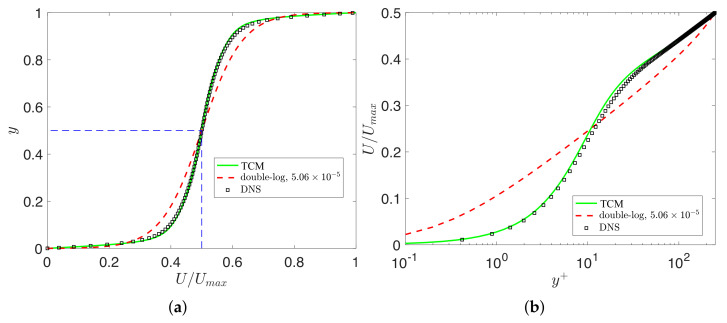
Turbulent Couette flow at $Re_{\tau }=250$: (**a**) “-”, TCM predictions; “- -”, best fit of the double-log profile in Equation (29) with $d=1.06\times 10^{-5}$; “□”, DNS data from [32]; (**b**) wall units scaling for the mean velocity profiles.

Here, we consider the predictions from the universal scaling fractional order $\alpha^{*}\left(y^{+}\right)$, and we also compare it against the double-log profile. The variable fractional order $\alpha^{*}\left(y^{+}\right)$ is between zero and one in our turbulence model. So, we work in the half-plane y∈[0,0.5] (see the dashed square in Figure 14a). We then obtain the results in the other half of the domain with U(y)=1−U(1−y),y∈(0.5,1]. Figure 14 shows the mean velocity profiles predicted using (29) and the mean velocity, which is predicted by the variable fractional order $\alpha^{*}\left(y^{+}\right)$. We can observe that the variable fractional model is in agreement with the experiment data as well as the double-log profile. However, the double-log profile is unable to capture the correct mean velocity near the wall. We also tested the profiles for low Reynolds number $Re_{\tau }=52$, where the numerical data were obtained from reference [31]. For the double-log profile, we could not find a suitable parameter *d* to obtain a good fit for the low $Re_{\tau }=52$. Finally, we show the comparisons between the TCM predicted mean velocities and DNS data at $Re_{\tau }=250$ obtained from reference [32]. Figure 15 shows that the fractional predictions are correct almost everywhere, especially near the wall regions for high Reynolds numbers.

### 3.4. Turbulent Boundary Layer Flow

**Figure 16:**
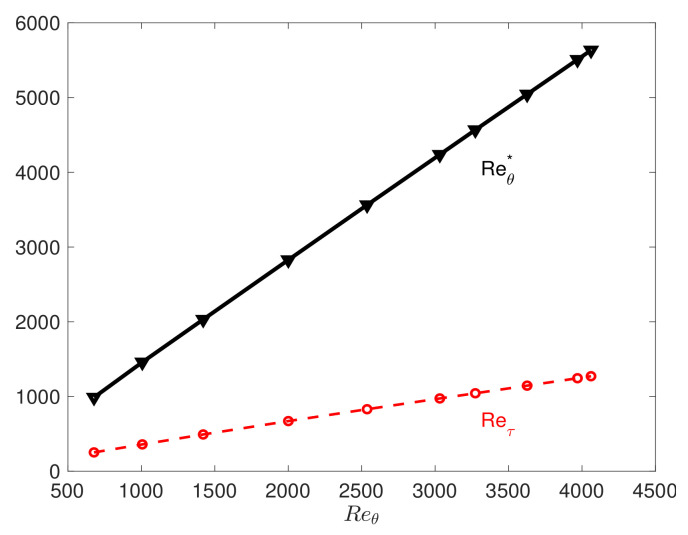
The relation between the friction Reynolds number $Re_{\tau }$ and $Re_{\theta }$.

In this subsection. we focus on the boundary layer problem. We use data available from the KTH turbulence group from the turbulent boundary layer DNS [34,35]. We first investigate the correlations between $Re_{\theta }$ (*x*-variable) and $Re_{\tau }$ (*y*-variable); Figure 16 shows the downstream variations in the friction Reynolds number $Re_{\tau }$, and unlike the channel flow, here, $Re_{\tau }$ is a function of the streamwise distance *x*.

**Figure 17:**
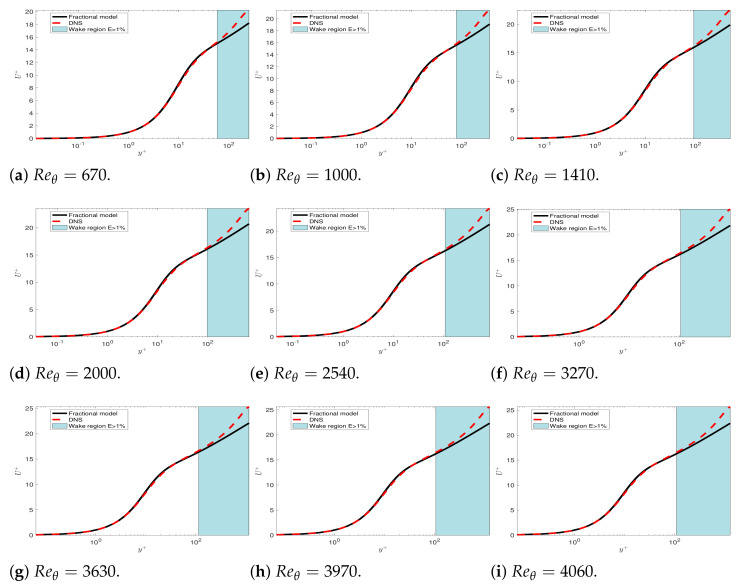
TCM: Boundary layer mean velocity profiles from the DNS and fractional modeling near the wall and in the wake region for several $Re_{\theta }$ from 670 to 4060.

**Figure 18:**
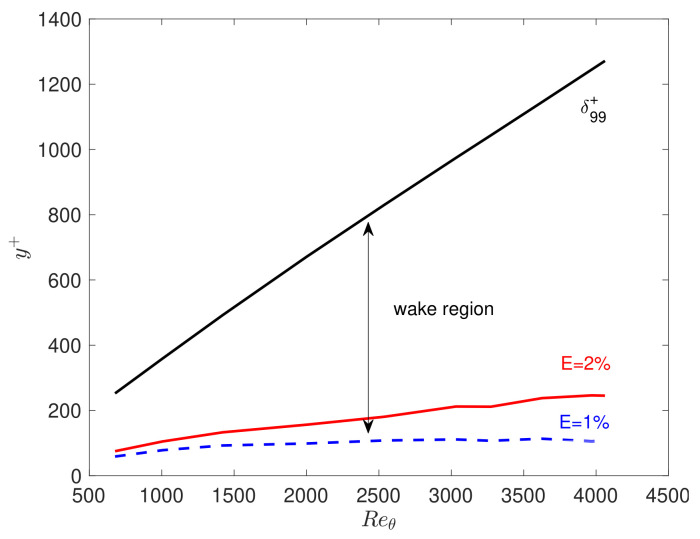
Downstream variations in $\delta_{99}^{+}$, and the error E=2% and E=1%. The lower bounds of the wake region are denoted by the blue curve with E=1% and the red curve with E=2% (see Equation (30)).

Then, we test if the mean velocity of the boundary layer problem exhibits any universality as the channel and pipe flow. We solve the forward boundary layer problem with the fractional order predicted by Equation (24) (i.e., the formula is the same as the channel flow case) including the wake region. Figure 17 presents the mean velocity profiles from the DNS [34] and fractional modeling near the wall for several $Re_{\theta }$ from 670 to 4060, with the corresponding $Re_{\tau }$ varying from 252 to 1200. We observe that the mean velocities are different in the wake region for different $Re_{\tau }$. Figure 18 plots the wake region, which is between $\delta_{99}^{+}$ and the error E=1%. We define this error as the difference in the mean velocity between the DNS data and the fractional model as follows:

(30) $$E=\frac{U-U_{f}}{U_{\infty }},$$

where *U* is the DNS data and $U_{f}$ presents the numerical results from the fractional model.

**Figure 19:**
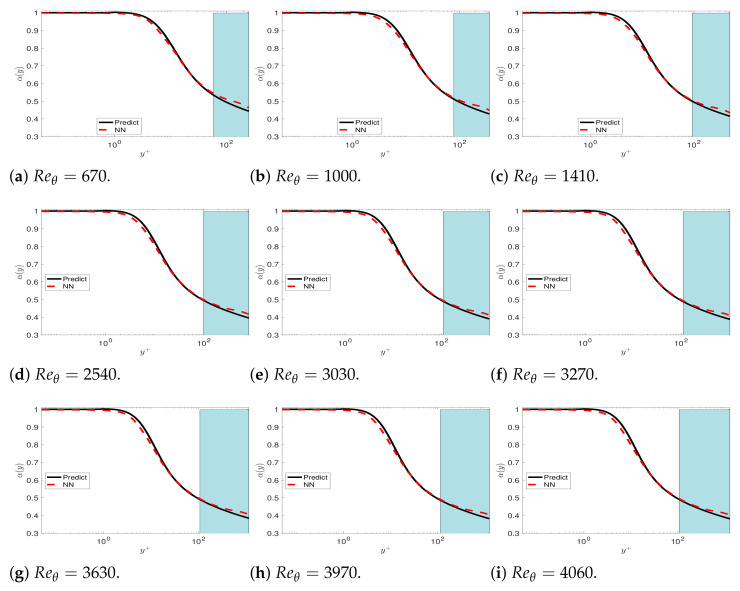
TCM: The fractional order α(y) learning from a neural network (NN) near the wall and in the wake region for several $Re_{\theta }$ from 670 to 4060, and the corresponding $Re_{\tau }$ from 252 to 1200. We can observe that the fractional order is different for different $Re_{\theta }$ in the wake region. The black line represents the reference fractional order predicted by channel flows; the red curve represents the NN results for different $Re_{\theta }$.

**Figure 20:**
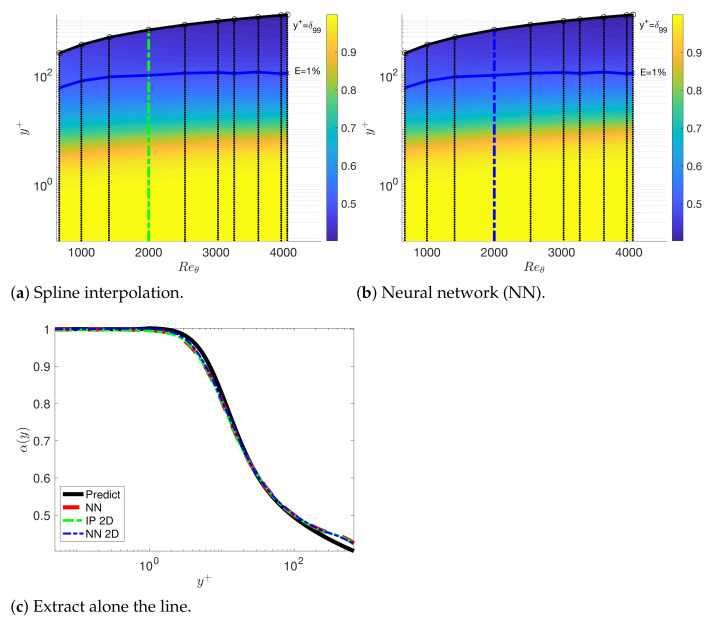
We train the fractional order in the wake region and near the wall selecting the data set $Re_{\theta }=670$ to 4060, excluding $Re_{\theta }=2000$; the training region is ($Re_{\theta }$, $y^{+}$)∈[670,4060]×[0,1200]. The training data set is represented as black dots: (**a**) we use spline interpolation (IP) in 2D; (**b**) the fractional order is trained by a neural network with 2 hidden layers and 20 neurons in each hidden layer. (**c**) The black line represents the reference fractional order predicted by channel flows; the red curve represents the fPINN results at $Re_{\theta }=2000$; the green line plots the interpolation results IP2D along the green line in (**a**); the blue curve presents the NN along the blue line in (**b**).

**Figure 21:**
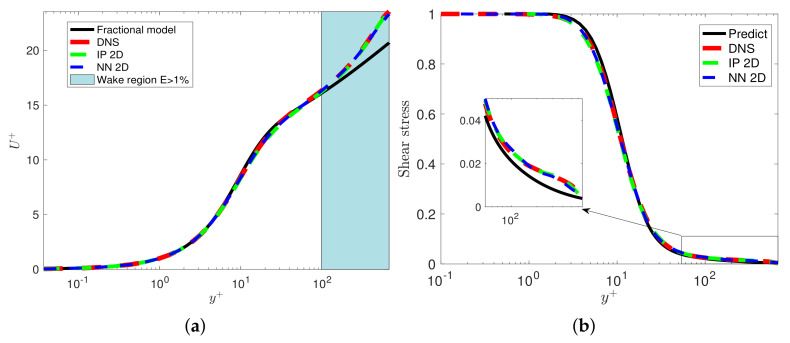
We solve the fractional turbulent boundary layer model with the fractional orders represented in Figure 20c at $Re_{\theta }=2000$. (**a**) The mean velocity; (**b**) the viscous shear stress. The black line represents the reference fractional order predicted by channel flows; the red curve represents the NN1D results at $Re_{\theta }=2000$; the green line plots the interpolation results IP2D for $Re_{\theta }$; the blue curve represents the NN for $Re_{\theta }=2000$.

Since the mean velocity does not exhibit universality in the wake region, we solve the fPINNs to investigate the variations in the fractional order in the wake region. In Figure 19, we plot the fractional order inferred by fPINN based on the DNS data for $Re_{\theta }=670$ to 4060. We can observe that the fractional order varies for different $Re_{\theta }$ in the wake region. Then, we train the fractional order in the wake region selecting the data set $Re_{\theta }=670$ to 4060 but excluding $Re_{\theta }=2000$. In Figure 20, we present the factional order in the 2D plane for the *x*-axis and $y^{+}$-axis. Finally, we solve the fractional turbulent boundary layer model with the fractional orders presented in Figure 20. The comparison between the numerical results and the DNS data set is presented in Figure 21.

## 4. Summary

We proposed multiple fractional models for wall-bounded turbulent flows in benchmark cases where the mean flow is either one-dimensional (channel, pipe, and Couette flows) or two-dimensional (boundary layer). The main idea is to employ a variable-order fractional gradient that depends on the distance from the wall, starting with an integer order at the wall. The computational problem we addressed is the discovery of the fractional variable-order profile given DNS or experimental data for the mean velocity profile. To this end, we formulated an inverse problem for the fractional order as a function of the distance from the wall, and we solved it using a finite difference method point-by-point and through a new fractional physics-informed neural network (fPINN) that encodes the physics of turbulence expressed via the fractional derivative of variable order. The fractional order is a function of the distance from the wall, a unique capability enabled by fractional calculus. We discovered that this fractional order function is universal for all Reynolds numbers and for different geometries.

The main contributions of this work are: (1) new fractional turbulent models with variable order are presented to model the total shear stress of RANS; (2) two solution methods for the non-trivial inverse problem, a FD method, and a fPINN for obtaining the fractional order function; (3) a universal fractional order profile was discovered for the channel and pipe flows that allowed us to accurately predict the fractional order for the boundary layer flows.

## Figures and Tables

**Table 1:** VFM-I: The history of the loss function with different training data sets for $Re_{\tau }=2000$. Log represents the log-uniform training points set.

Itr	$N_{t}=500$	$N_{t}=1000$	$N_{t}=2000$	Log, $N_{t}=10$	$N_{t}=20$	$N_{t}=40$	$N_{t}=80$
0	$6.08\times 10^{-1}$	$4.70\times 10^{-1}$	$6.57\times 10^{-1}$	$6.92\times 10^{-1}$	$7.01\times 10^{-1}$	$6.74\times 10^{-1}$	$6.90\times 10^{-1}$
5000	$1.04\times 10^{-4}$	$8.11\times 10^{-5}$	$9.12\times 10^{-5}$	$4.72\times 10^{-1}$	$5.94\times 10^{-5}$	$5.08\times 10^{-5}$	$4.61\times 10^{-5}$
10,000	$1.79\times 10^{-5}$	$1.32\times 10^{-5}$	$1.21\times 10^{-5}$	$8.27\times 10^{-6}$	$8.93\times 10^{-6}$	$1.08\times 10^{-5}$	$9.51\times 10^{-6}$
20,000	$3.34\times 10^{-6}$	$1.75\times 10^{-5}$	$1.40\times 10^{-6}$	$9.26\times 10^{-7}$	$4.68\times 10^{-7}$	$2.84\times 10^{-6}$	$2.77\times 10^{-6}$
30,000	$2.41\times 10^{-6}$	$7.31\times 10^{-7}$	$7.12\times 10^{-7}$	$4.41\times 10^{-7}$	$2.05\times 10^{-7}$	$1.55\times 10^{-6}$	$2.08\times 10^{-6}$

## Data Availability

Not applicable.
